# Composition, interaction networks, and nitrogen metabolism patterns of bacterioplankton communities in a grassland type Lake: a case of Hulun Lake, China

**DOI:** 10.3389/fmicb.2023.1305345

**Published:** 2023-11-22

**Authors:** Yujiao Shi, Wenbao Li, Xin Guo

**Affiliations:** ^1^Water Conservancy and Civil Engineering College, Inner Mongolia Agricultural University, Hohhot, China; ^2^Inner Mongolia Key Laboratory of Protection and Utilization of Water Resources, Hohhot, China

**Keywords:** water ecosystems, co-occurrence network, potential function, community structure, environmental preferences

## Abstract

The composition of bacterial communities in freshwater ecosystems is influenced by numerous factors including environmental conditions and biological interactions. In grassland inland closed lakes, factors affecting lake ecosystems are either exogenous or endogenous, contributing to the formation of distinct habitats in the surface and bottom waters of the bacterial communities. However, the extent to which environmental factors selectively shape the bacterial communities in aquatic systems remains unclear. Therefore, we sampled the surface, middle, and bottom waters at 13 sampling points in each layer. High-throughput sequencing techniques were employed to examine the spatial heterogeneity of the bacterial community structure during summer in Hulun Lake, the largest grassland-type lake in Inner Mongolia, China, to determine the microbial community dynamics and symbiosis patterns under different habitat conditions. Our results revealed a decrease in the diversity and heterogeneity of the bacterioplankton community, influenced by changes in the environment from exogenous inputs to endogenous releases. Furthermore, this alteration in community structure was concomitant with enhanced co-occurrences among microorganisms in the bottom water layers. This finding suggests that endogenous release promotes heightened symbiotic interactions, thereby facilitating the development of more complex modular structures. Symbiotic networks in different layers were differentiated by key species, with the ecological clustering modules of these species demonstrating dissimilar environmental preferences. The microbial communities were highly habitat-specific, mimicking responses to total nitrogen (TN) in the surface layer, pH in the middle layer, and chemical oxygen demand (COD) in the bottom layer. Bacterioplankton functions were assessed using Tax4Fun, indicating exogenous inputs and endogenous release increased the relative abundance of genes with nitrogen-fixing and nitrification potential nitrogen metabolism functions in surface and bottom waters, respectively. With Planctomycetota and Proteobacteria phyla as potential key groups for regulating nitrogen metabolic processes, Proteobacteria may facilitate the depletion of nitrate in surface and bottom waters, while the close contact of surface waters with the atmosphere accelerated Planctomycetota-dominated nitrogen fixation into the lake. Our findings contribute to the understanding of vertical microbial diversity and its network patterns in grassland type lakes, underscoring the potential role of environmental factors (exogenous inputs and endogenous releases) in bacterioplankton community formation.

## 1 Introduction

Among the diverse life forms on Earth, bacteria are characterized by their taxonomic diversity, ubiquitous distribution, and intricate interaction networks. They play a crucial role in mediating metabolic processes within biogeochemical cycles, thereby significantly contributing to ecosystem stability and ecological functionality (Liu et al., 2021; Wu et al., 2021). Bacterioplankton serve not only as an integral component of lake ecosystems but also as a key indicator for water quality assessment (van de Graaf et al., 1995; Karpowicz et al., 2012; Shang et al., 2022). They facilitate the efficient functioning of lake ecosystems by participating in the cycling of chemical elements, such as nitrogen, within the water column (Tammert et al., 2015; Grover, 2017; Gu et al., 2020; Wang Y. B. et al., 2020). Various studies have demonstrated that bacterioplankton community structure is affected by different environmental indicators. De Menezes et al. (2015) discovered a relationship between community composition and variables such as moisture, and nitrogen. Zhang L. Y. et al. (2021) suggested that community diversity significantly affects total nitrogen (TN). Wahdan et al. (2023) identified a correlation between the relative abundance of microorganisms and chemical composition. These findings indicate that the use of bacteria as indicators for monitoring the evolution of aquatic environments is highly sensitive to environmental changes and has substantial implications for the health of lake ecosystems (Swift et al., 1979; Bertilsson et al., 2007; Dumbrell et al., 2010).

Lakes in Inner Mongolia serve as critical water resources in the ecologically vulnerable regions of northern China, fulfilling essential roles in economic development, ecological balance, and environmental conservation (Li et al., 2019). Hulun Lake, the largest lake in Inner Mongolia, is representative of grassland-type lakes in the high-latitude, cold, and arid regions. It is vital for climate regulation, water resource protection, desertification prevention, and the maintenance of grassland ecosystem equilibrium (Zhang et al., 2020). Because of its unique geographical location, the primary nutrient sources for the lake are dried grass from the expansive Hulunbeier grassland, municipal wastewater, and animal feces, mainly from cattle and sheep (Chen et al., 2021). These nutrients are transported into the lake by rivers and winds, resulting in what is known as exogenous inputs, and these exogenous inputs lead to increased levels of organic pollutants in the surface waters. It documented that Hulun Lake has been in a state of eutrophication since the 1980s (Wang, 2006; Michael and Jörg, 2008; Chuai et al., 2012; Quiza et al., 2014). In addition, extensive research has indicated that sediments act as a “source” of nutrients in the water column. Nutrients stored in sediments are continuously released into upper water layers (Nikolai and Dzialowski, 2014; Zhang H. G. et al., 2021). This endogenous release of sediments makes the environment stratified between the bottom and surface layers (Zhang et al., 2016; Dong et al., 2019; Khabouchi et al., 2020; Wang et al., 2023). Therefore, these aquatic ecosystems provide an optimal option for investigating environmental sensitivities and habitat preferences of bacterial communities.

Although incremental progress has been made in studies concerning the distribution patterns of elements and bacterioplankton in eutrophic grassland-type lakes, such as Hulun Lake (Wang et al., 2018; Zhang et al., 2019; Shang et al., 2020), existing research largely lacks mechanistic explanations for how the bacterioplankton community responds to variations in the aquatic environment attributable to exogenous inputs and endogenous releases. This gap presents a considerable challenge for ecologists aiming to establish ecological criteria for lakes, particularly with respect to interactions between nutrients and microorganisms. Therefore, an in-depth analysis of the dynamics and structure of the bacterioplankton community in Hulun Lake, as an integral component of the aquatic ecosystem, would yield valuable insights for more effective freshwater lake management (Dimitry et al., 2014).

To address these knowledge gaps, in this study, we investigated bacterial diversity, environmental drivers, and nitrogen functioning patterns in aquatic systems influenced by both exogenous inputs and endogenous releases. Moreover, we examined the symbiotic relationships within bacterial communities. Specifically, within the context of grassland-confined inland lakes affected by both exogenous inputs and endogenous releases, three research questions were posed. (1) Do bacterial communities in the surface, middle, and bottom water columns exhibit differences in diversity and structure? (2) Do different environmental drivers affect the structure and nitrogen metabolism patterns of the coexisting bacterial communities? (3) Do symbiotic relationships and associated environmental preferences among these bacterial communities vary across different water layers?

Building on the aforementioned knowledge gaps, this study focused on Hulun Lake as the study area. Using high-throughput sequencing technology, we investigated the response relationships between environmental shifts caused by both exogenous inputs and endogenous releases, and the micro-ecological system of the lake. Employing a stratified sampling approach during the summer months, we combined changes in typical physicochemical indicators and nutrient elements across the surface, middle, and bottom water layers. This facilitated examination of the structural evolution of the dominant phyla and genera in the bacterioplankton community at varying depths. Additionally, we explored variations in the ecological structure of these bacterioplankton communities. The aim was to promote limited micro-ecological studies on Hulun Lake while providing a theoretical foundation for understanding its environmental evolution.

## 2 Materials and methods

### 2.1 Study lake

**Figure 1:**
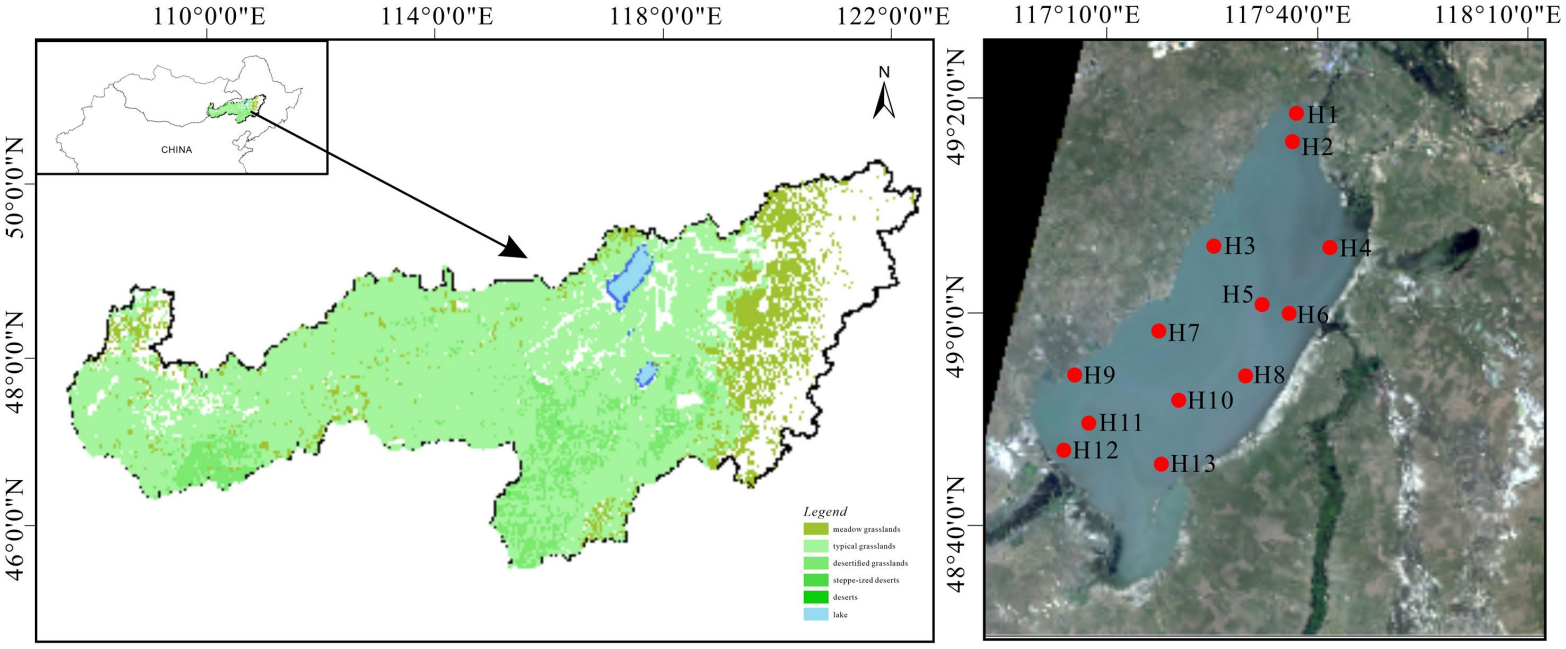
Geographical location of Hulun Lake and sampling site distribution.

Hulun Lake (116°58′–117°48′E, 48°33′–49°20′N) is located in Hulunbeier City in northern China and southern Mongolia, across Beier Lake. It lies 57 km from the China–Mongolia western border and 15 km from Russia to the north, which is at the junction of China, Mongolia, and Russia. Recognized as the premier freshwater lake in northern China, Hulun Lake is a unique natural asset with biodiversity and ecological multifunctionality unparalleled globally in cold and arid regions (Zhang et al., 2017; Li et al., 2020). Its topography is irregularly sloping and elongated, flanked by expansive grasslands and a flat terrain. It covers an approximate surface area of 2,043 km^2^ and exhibits climatic characteristics such as a multi-year average temperature of −0.60°C, precipitation of 352.30 mm, evaporation of 1549.80 mm, and wind speed of 3.45 m/s (Li et al., 2019; Wang W. L. et al., 2020). Influenced primarily by the mid-temperate continental monsoon climate, the region experiences pronounced seasonal variability, including short spring and summer seasons and prolonged winter. Precipitation is concentrated from late June to early August (Zhao et al., 2017). Regarding water recharge, the lake primarily receives inflows from the Kelulun River, Wuerxun River, and Hailaer River, in addition to precipitation (Figure 1). The water supply is crucial for the preservation of grassland biodiversity and ecological integrity (Li et al., 2019).

### 2.2 Sample collection

Prior to the main study, a preliminary survey was conducted to identify 13 sampling sites (H1–H13) arranged from north to south for summer water sample collection in August 2021 covering the entire lake. Water samples were collected at three different depths: upper (0–15 cm below the water surface), middle (half the depth of the water column), and lower (10–50 cm above the sediment–water interface). The samples were collected using a water extraction device with a 1 L sterile polyethylene bottle employed as the collection device. Pre-disinfected with ultrapure water and alcohol, each bottle was rinsed three times with water from the respective sampling point before sample collection. Three bottles were secured from each point, refrigerated, and promptly transported to the laboratory for analysis. Concurrent with sample collection, a multi-parameter water quality monitor was used to measure various physicochemical indicators. These examples were used for the determination of nutrients in the water column and collection of bacterioplankton.

### 2.3 Measurement of environmental factors

At each sampling site, key physicochemical parameters, namely water temperature (WT), electrical conductivity (EC), pH, dissolved oxygen (DO), salinity (SAL), oxidation–reduction potential (ORP), and total dissolved solids (TDS), were measured using a multi-parameter water quality monitor. Laboratory analyses were conducted to quantify nutrient concentrations, including free-state ammonia nitrogen (NH_4_^+^-N), TN, chlorophyll-a (Chla), total phosphorus (TP), dissolved inorganic phosphorus (DIP), total dissolved phosphorus (TDP), and total organic matter (COD), in accordance with standard methods.

### 2.4 Sample processing, DNA extraction, and PCR amplification

Genomic DNA from microbial communities was extracted from water samples using a DNA Kit (Omega Bio-tek, Norcross, GA, United States) according to the manufacturer’s instructions. DNA quality and concentration were assessed using a 1% agarose gel and quantified using a NanoDrop 2000 UV–Vis spectrophotometer (Thermo Scientific, Wilmington, United States). The V3–V4 hypervariable region of the bacterial 16S rRNA gene was amplified using primers 338F (5′-ACTCCTACGGGAGGCAGCAG-3′) and 806R (5′-GGACTACHVGGGTWTCTAAT-3′) on an ABI GeneAmp^®^ 9700 PCR thermocycler (ABI, CA, United States) (Walters et al., 2016). PCR amplification of the 16S rRNA gene was performed using the following thermal profile: initial denaturation at 95°C for 3 min, followed by 27 cycles of denaturation at 95°C for 30 s, annealing at 55°C for 30 s, and extension at 72°C for 45 s, followed by a final extension at 72°C for 10 min, and termination at 4°C. The PCR mixture was composed of 4 μL of 5 × TransStart FastPfu buffer, 2 μL of 2.5 mM dNTPs, 0.8 μL each of 5 μM forward and reverse primers, 0.4 μL of TransStart FastPfu DNA Polymerase, 10 ng of template DNA, and ddH_2_O to achieve a final volume of 20 μL. The reactions were performed in triplicates. After amplification, the PCR products were extracted from a 2% agarose gel, purified using an AxyPrep DNA Gel Extraction Kit (Axygen Biosciences, Union City, CA, United States) according to the manufacturer’s instructions, and quantified using a Quantus^™^ Fluorometer (Promega, United States).

### 2.5 Illumina MiSeq sequencing

Purified amplicons were pooled in equimolar ratios and subjected to paired-end sequencing on an Illumina MiSeq PE300 or NovaSeq PE250 platform (Illumina, San Diego, CA, United States), based on the standard protocols provided by Majorbio Bio-Pharm Technology Co. Ltd. (Shanghai, China).

### 2.6 Processing of sequencing data

Raw 16S rRNA gene sequencing reads were demultiplexed and quality-filtered using fastp version 0.20.0 (Chen et al., 2018), and subsequently merged with FLASH version 1.2.7 (Mago and Salzberg, 2011). The quality filtering and merging criteria were as follows: (i) reads of 300 bp were truncated if any site displayed an average quality score lower than 20 across a 50 bp sliding window, if truncated reads were shorter than 50 bp, or if reads containing ambiguous characters, they were discarded; (ii) only overlapping sequences exceeding 10 bp were assembled based on their overlapping regions, with a maximum mismatch ratio set at 0.2. Unassembled reads were discarded; and (iii) samples were distinguished by barcode and primer sequences, with sequence direction adjusted accordingly, employing exact barcode matching and allowing for a two-nucleotide mismatch in primer matching.

Operational taxonomic units (OTUs) were clustered using UPARSE version 7.1 (Edgar, 2013) with a 97% similarity cut-off (Stackebrandt and Goebel, 1994; Edgar, 2013), and chimeric sequences were removed. Taxonomic analysis for each representative OTU sequence was performed using the RDP Classifier version 2.2 (Wang et al., 2007) against the 16S rRNA database (e.g., Silva v138), using a confidence threshold of 0.7.

### 2.7 Data analysis and statistics

Alpha diversity indices, including Good’s coverage, Chao1, Shannoneven, Shannon and phylogenetic diversity, were evaluated at a 97% OTU similarity level using mothur (version v.1.30.2). Venn diagram analysis was performed using R language (version 3.3.1) for statistical analysis and graphing. Community composition differences were assessed using the Kruskal-Wallis rank sum test, while beta diversity distance matrices were computed using Qiime. NMDS plots based on Bray-Curtis distances were generated using the R language (version 3.3.1) vegan package. Distance redundancy analysis (RDA) and Mantel tests were conducted for variance classification and genus abundance data assessment, respectively. These analyses were performed using the “vegan” package in R. The genus and environmental variables were fitted to ordination plots to assess the relative significance of each environmental factor in elucidating community variation using the goodness-of-fit statistic (R^2^) (*p* < 0.05). To reduce the complexity of the ecological network, only the top 500 genera were selected based on their relative abundance across different water depths. Network metrics, such as mean node degree, clustering coefficient, mean path length, modularity, network density, and network diameter, were calculated and visualized using Gephi (version 0.9.2). Subsequent analyses were conducted to identify the microbial modules in the underlying water and their association with water quality metrics. Following log-transformation and normalization of the environmental variables, Spearman correlations were calculated, considering *p* < 0.05, as indicative of a valid relationship. Taxonomic profiling was performed by converting 16S rRNA sequences based on the Silva database into the corresponding prokaryotic profiles in the KEGG database. The normalization of prokaryotic abundance in the corresponding KEGG database was performed using the 16S copy numbers. Finally, we employed homogenized abundance data and combined the correspondence between the prokaryotes in the KEGG database and the 16S taxonomic profiles in the Silva database to identify and quantify the different functional genes involved in nitrogen metabolism within the microbial communities. To assess the impact of dominant phyla on these functional genes, correlation analyses were conducted to determine the correlation between both using *p*-values. All statistical analyses were performed using Origin version 2023.

## 3 Results

### 3.1 Environmental characterization

**Figure 2:**
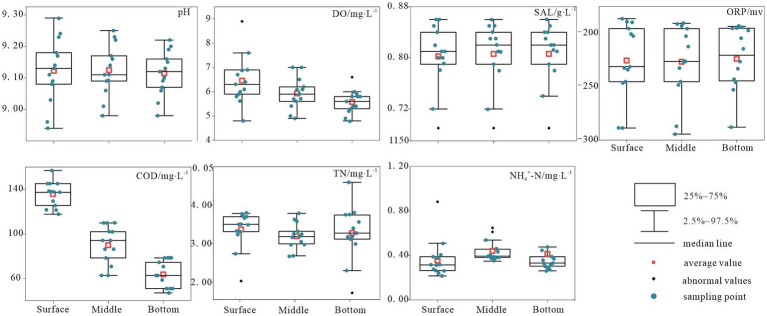
Variation characteristics of typical physicochemical indices in Hulun Lake.

Figure 2 presents the comprehensive environmental parameters of Hulun Lake water sampled at various depths. Owing to the influence of multiple factors, the physicochemical properties of the surface, middle, and bottom water samples from the Hulun Lake varied to some extent. By measuring seven indicators, including physicochemical and nutrient elements, the results revealed that pH and TN concentrations were the highest in the surface water of the lake (mean values of approximately 9.12 and 3.37 mg/L, respectively) and the lowest in the middle water of the lake (mean values of approximately 9.11 and 3.19 mg/L, respectively). The ORP levels increased from the surface to the bottom, reaching a peak mean value of approximately −223.93 mV in the bottom layer. Conversely, DO and COD demonstrated a declining trend from surface to bottom, with minimum mean values of 5.58 mg/L and 88.41 mg/L, respectively, in the bottom layer. The SAL remained relatively stable in the middle and bottom layers, but was lowest in the surface layer, with a mean value of approximately 0.81 mg/L. Overall, horizontal variations were observed in the physicochemical indicators. Specifically, NH_4_^+^-N in the middle water column exhibited significant fluctuations and outlier values, whereas the remaining indicators exhibited relatively stable distributions (Figure 2).

### 3.2 Abundance and α-diversity of bacterioplankton in Hulun Lake

To elucidate the variations in bacterial communities across different depths in the water column of Hulun Lake, we employed NMDS maps to depict species composition similarities. Additionally, the Chao, Shannoneven, and Shannon indices were utilized to rigorously examine community richness, evenness, and diversity in alpha diversity. These analyses provided insights into the distribution characteristics of bacterioplankton communities in the surface, middle, and bottom water layers of Hulun Lake.

**Figure 3:**
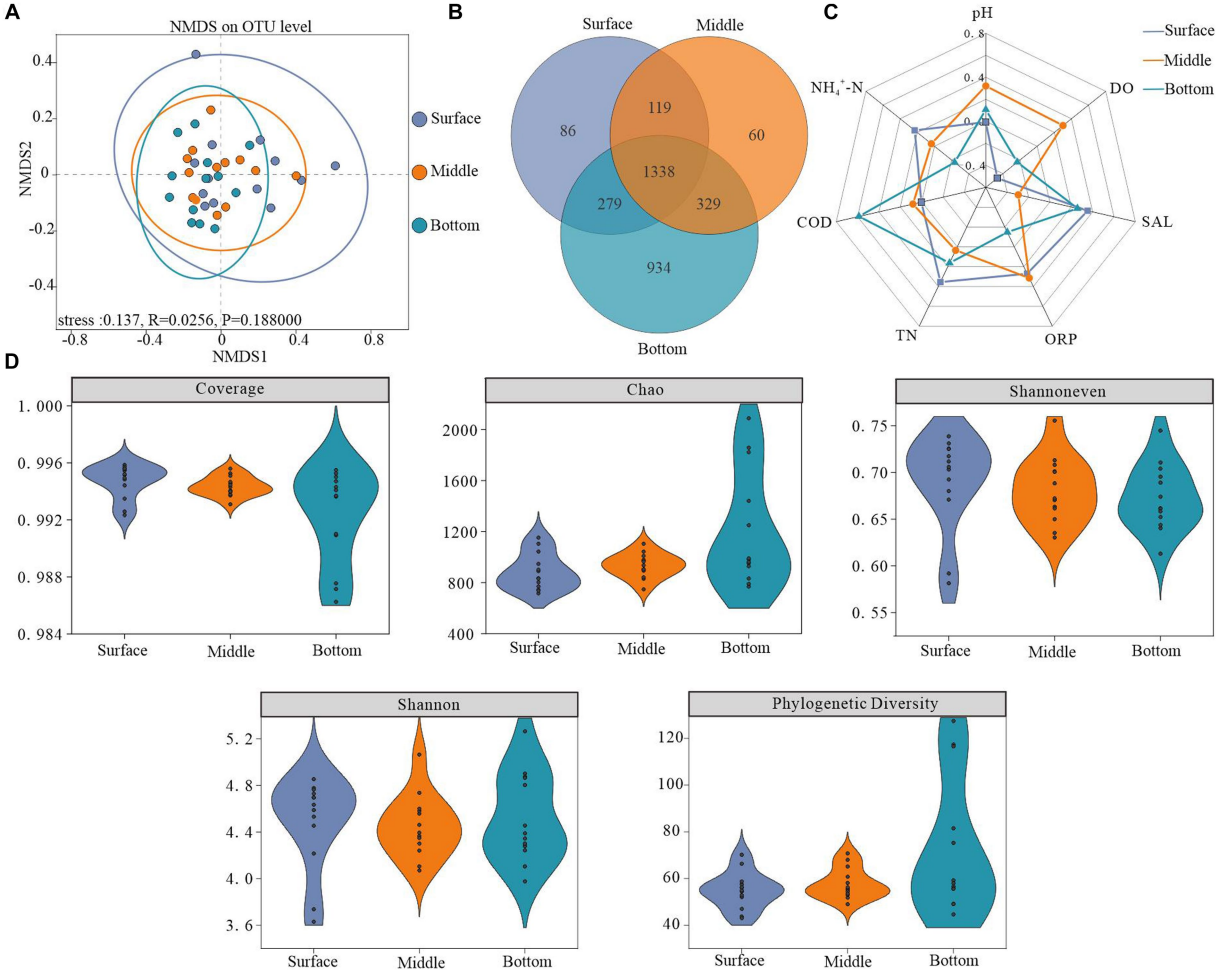
Microbial diversity across different depths. **(A)** Non-metric multidimensional scaling analysis (NMDS) based on surface, midwater, and bottom bacterioplankton communities at the OTU level (using the Bray–Curtis dissimilarity matrix), **(B)** number of shared and unique OTUs at different depths, **(C)** radar plot showing factors significantly correlated with bacterioplankton diversity (Pearson’s rank correlation, *p* < 0.05), and **(D)** bacterioplankton diversity indices.

NMDS analysis utilizing the Bray-Curtis distance algorithm demonstrated depth-dependent horizontal heterogeneity in water samples. Notably, variations within bacterioplankton communities decreased along a gradient from the surface layer to the bottom layer. Furthermore, community dispersion was significantly lower in the bottom layer than in the other two layers, accompanied by an increase in both community similarity and overall structure with increasing depth. The vertical variability in the structure of the bacterioplankton community indicated that the water depth had a profound influence on the structure of the bacterioplankton communities (Figure 3A).

The Coverage index values for all sample libraries exceeded 99%, indicating that the generated sequences adequately represented microbial communities at the study sites. Higher values of the Chao, Shannoneven, and Shannon indices, representing alpha diversity characteristics, demonstrated higher abundance, homogeneity, and diversity within the bacterioplankton communities. As depicted in Figure 3D, a depth-dependent increase in the Chao index was observed, indicating the lowest bacterioplankton abundance in surface water and the highest abundance in bottom water. Conversely, the lowest Shannoneven and Shannon indices were recorded in the bottom and middle water, respectively. These results suggest that while the bottom layer exhibited high bacterial abundance but an uneven distribution, the middle layer demonstrated the lowest diversity. Interestingly, phylogenetic diversity indices exhibited minor depth-dependent variations and shared OTUs constituted 42.5% of the total OTUs (Figures 3B,D).

Our analysis revealed a notable depth-dependent evolution of the environmental factors affecting bacterioplankton diversity. Specifically, microbial diversity in the surface layer exhibited a strong negative correlation with DO, whereas COD positively influenced the diversity in the bottom layer (Figure 3C; Supplementary Table S1).

### 3.3 Variation in the response of bacterioplankton composition to lake depth and environmental drivers

**Figure 4:**
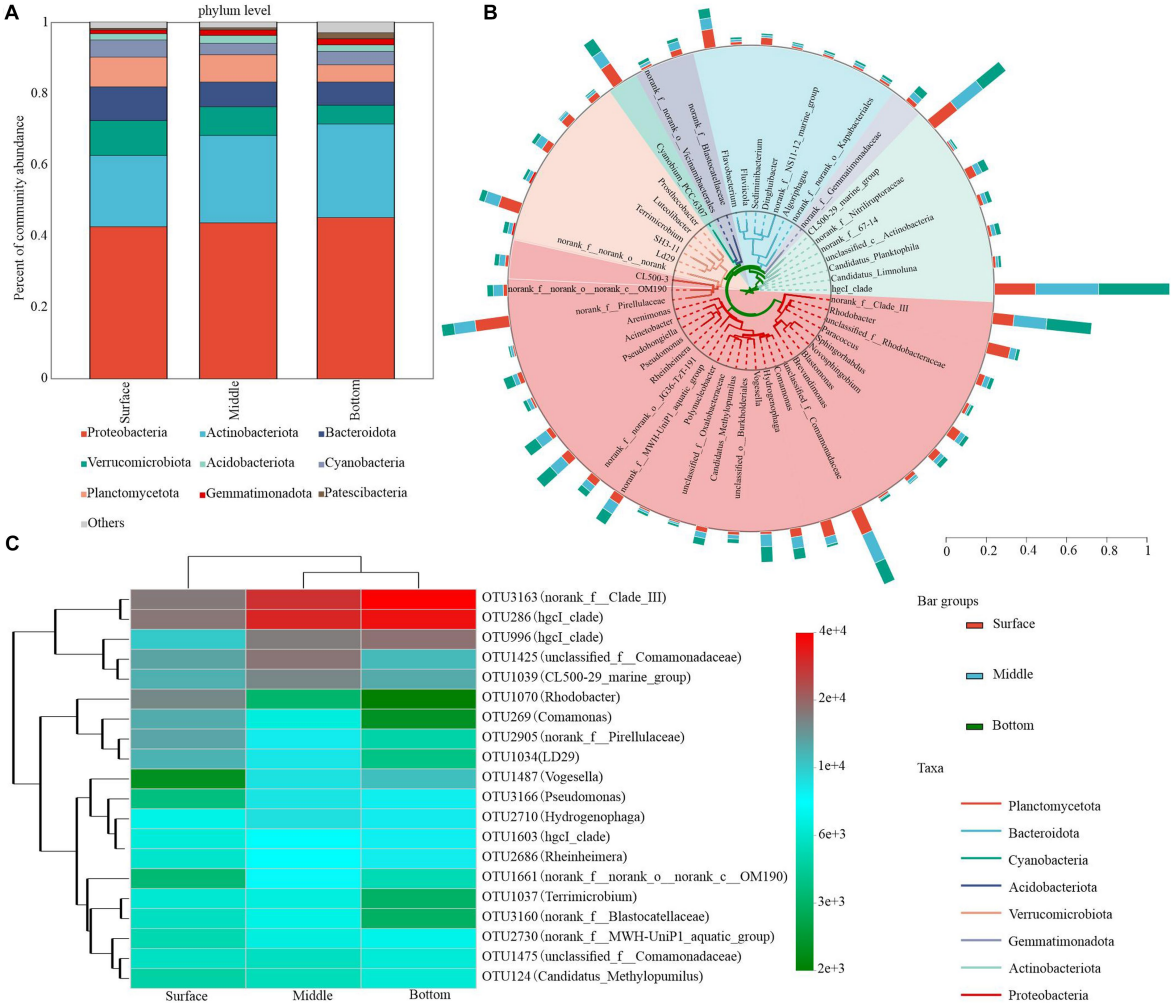
Bacterial community composition in water at different depths. **(A)** Phylum level (relative abundance >1%). **(B)** Phylogenetic tree showing the top 50 genera with the highest relative abundances. Taxonomic affiliations of each genus at the phylum level were determined by the color range within the tree. **(C)** Heatmap depicting the dynamics of the top 20 asv species classified at the genus level in the bottom water column of the table.

The bacterial community composition plays a pivotal role in nutrient cycling and energy transfer in water. Upon collecting and analyzing samples from the surface, middle, and bottom water layers, 10 dominant bacterial phyla were detected in samples where the relative abundance exceeded a 1% threshold. Proteobacteria and Actinobacteria constituted the majority of bacterioplankton OTUs, accounting for a combined relative abundance exceeding 50% across all depths (approximately 42.00 and 21.20% in surface water, 44.70 and 27.50% in midwater, and 49.20 and 30.80% in bottom water, respectively). In contrast, the relative abundance of other dominant phyla, such as Verrucomicrobiota, Bacteroidetes, and Planctomycetota, declined progressively from 11.20, 6.61, and 10.60% in the surface layer to 3.40, 3.13, and 6.26% in the bottom layer, respectively (Figure 4A). Patescibacteria emerged as the most dominant bacterial group, demonstrating a significant depth-dependent variability (Supplementary Table S1).

The dominant bacterial phylum was primarily dominated by five phylotypes with high relative abundance: *hgcI_clade* (attributed to Actinobacteriota), *norank_f__Clade_III* (attributed to Proteobacteria), *CL500-29_marine_group* (attributed to Actinobacteriota), *unclassified_f__Comamonadaceae* (attributed to Proteobacteria), and *norank_f__Pirellulaceae* (attributed to Planctomycetota). Although their relative abundance consistently changed with the depth of the lake, *hgcI_clade*, *norank_f__Clade_III*, *Rhodobacter*, and *Pseudomonas* exhibited the most pronounced changes. Specifically, the relative abundances of *hgcI_clade* and *norank_f__Clade_III* in the surface waters were 8.43 and 3.97%, respectively, which were significantly lower than those in the middle (13.09 and 6.22%, respectively) and bottom waters (14.66 and 8.46%, respectively). The relative abundance of *hgcI_clade* and *norank_f__Clade_III* increased with water depth, whereas the relative abundance of *norank_f__Pirellulaceae* declined from 6.42 to 2.33% in the surface layer. *CL500-29_marine_group* and *unclassified_f__Comamonadaceae* demonstrated minimal variation and peaked in the middle layer with approximate values of 6.16 and 5.80%, respectively (Figure 4B; Supplementary Table S2). Additionally, we observed that the highest OTUs in relative sequence abundance exhibited considerable variation with increasing lake depth. According to the cumulative relative abundance of the top 20 OTUs, the bacterioplankton community was predominantly characterized by OTU3163 and OTU286. Notably, dominant OTUs exhibited depth-related shifts. For instance, the third most dominant OTU in surface waters shifted from OTU1070 to OTU1425 and OTU996 in middle waters (Figure 4C; Supplementary Table S3).

The relative abundance of dominant microbial species across various layers is closely associated with the trophic status of the lake. In the surface and midwater layers, the dominant phyla, genera, and OTUs exhibited similar environmental responses. pH and TN were the primary factors affecting surface bacterioplankton, whereas pH, SAL, and DO were key determinants of midwater bacterioplankton (Supplementary Figure S1). Conversely, the environmental responsiveness of species at different taxonomic levels in the bottom layer exhibited greater variability. Specifically, the relative abundance of the dominant phyla was primarily influenced by pH, NH_4_^+^-N, and COD. The dominant genera and OTUs were mainly influenced by pH, NH_4_^+^-N, ORP, and SAL (Supplementary Figure S1).

The dominant species exhibited similar environmental responsiveness in both the surface and midwater layers. However, in the bottom layer, COD was positively correlated with Verrucomicrobiota, whereas ORP and NH_4_^+^-N were negatively correlated with the dominant genera. Additionally, pH and SAL were positively correlated with *unclassified_f__Comamonadaceae* and *hgcI_clade* (Supplementary Figure S1). These findings suggest that both external inputs and internal releases influenced not only the composition of the bacterioplankton community but also its interaction with environmental factors.

### 3.4 Vertical shifts in the ecological network structure of bacterioplankton

Network analysis not only provides an excellent exploration of potential interactions among microbial communities but also elucidates the complexities of community structure. Therefore, in this study, the ecosystem network structures of bacterioplankton in the surface, midwater, and benthic layers were constructed based on the top 500 genera and their relative abundances. The results revealed that the surface water network comprised 12,241 edges, with a graph density of 0.098. In contrast, the bottom water network contained 23,093 edges, with a higher graph density of 0.184. However, the middle water network had 10,078 edges and a lower graph density of 0.08, indicating less interconnectivity among the bacterioplankton communities (Supplementary Table S4).

**Figure 5:**
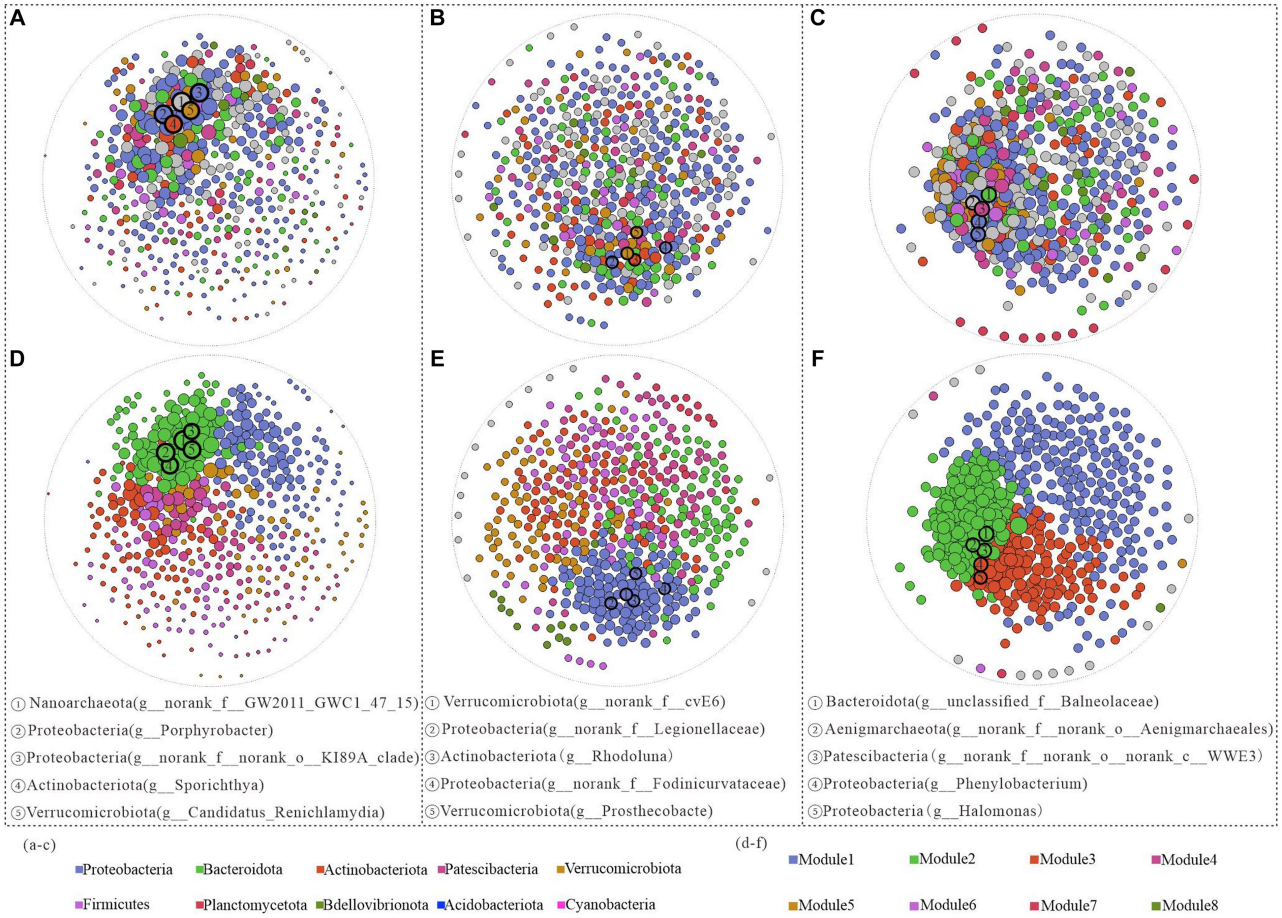
Network structure analysis of Hulun Lake based on genus level at different depths. Surface **(A)**, middle **(B)**, and bottom **(C)** water nodes are colored by phylum taxa, and surface **(D)**, middle **(E)**, and bottom **(F)** nodes are colored by module.

In the surface water samples, Nanoarchaeota (*g_norank_f_GW2011_GWC1_47_15*) exhibited the highest structural contribution and decreased structural contribution in the middle and bottom samples, indicating its pivotal role in stabilizing the bacterial community structure in the surface water (Figure 5A). In the middle water, other phyla, such as Actinobacteriota and Verrucomicrobiota, had greater network contributions than those in the bottom water. In particular, despite their low relative abundances in the bottom water, both Aenigmarchaeota (*g_norank_f_norank_o_Aenigmarchaeales*) and Patescibacteria (*g_norank_f_norank_o_norank_c_WWE3*) made substantial contributions to the ecological network structure (Figures 5B,C).

Subsequent modularization of all network nodes revealed a gradual increase in the number of modules from the surface to the bottom layers, along with variations in the keystone genera across different network structures. From the positions of the modules where all keystone genera were located in both the surface and midwater layers, the top five keystone genera were situated within the same module, but exhibited stronger interactions with each other in the surface layer than in the midwater network (Figures 5D,E). In contrast, the bottom water keystone genera were distributed across distinct modules. Specifically, Bacteroidota (*g_unclassified_f_Balneolaceae*), Aenigmarchaeota (*g_norank_f_norank_o_Aenigmarchaeales*), and Patescibacteria (*g_norank_f_norank_o_norank_c_WWE3*) were located on Module2, while Proteobacteria (*g_Phenylobacterium* and *g_Halomonas*) were situated in Module3. These patterns potentially reflect differences in ecological functionality among these genera (Figure 5F).

**Figure 6:**
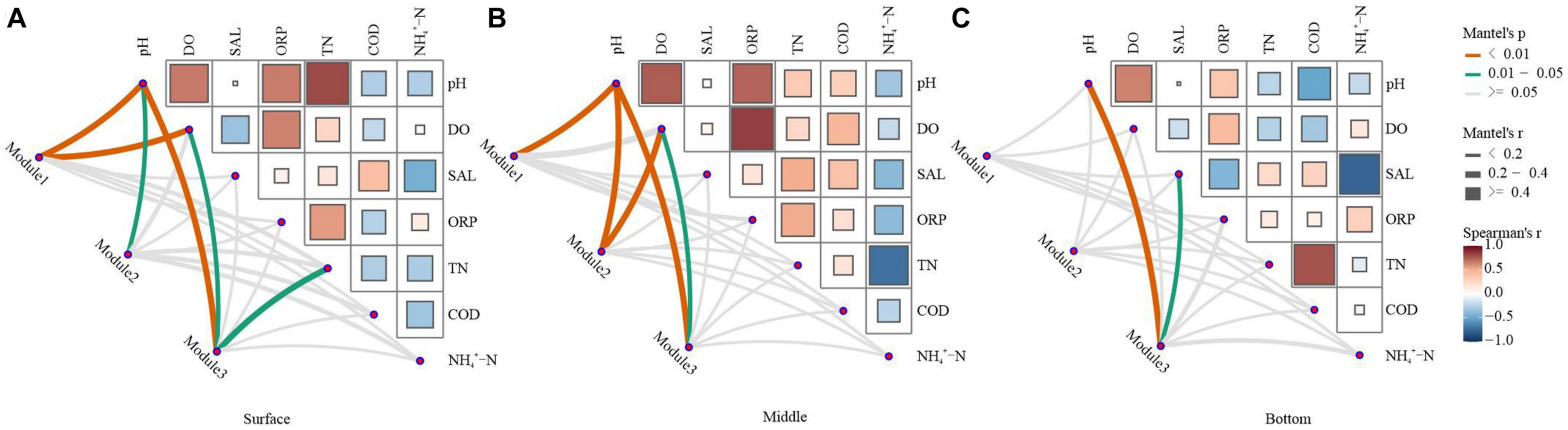
Environmental drivers of ecological network modules in water. The top three modules in the ecological network structure, which accounted for over 60% of the total number of modules, were selected and used as key modules to characterize the ecological network structure. Relationships between the surface **(A)**, middle **(B)**, and bottom **(C)** bacterial network modules and the physicochemical properties of water were calculated by Mantel test analysis. The edge width corresponds to the *R*-value, and the edge color indicates statistical significance. Color gradients indicate Pearson correlation coefficients between water physicochemical properties.

As observed in Figure 6, the correlation strength between the network structure and environmental variables was lower in the bottom water of the lake than in the surface and middle waters. In summary, bacterioplankton network modules at varying depths in Hulun Lake exhibited distinct associations with surveyed environmental factors. In the surface layer, Module1 was predominantly influenced by pH and DO, Module2 was significantly affected only by pH, and Module3 was notably impacted by pH, DO, and TN (Figure 6A). In the midwater layer, pH and DO emerged as principal factors shaping the network modules (Figure 6B). In the bottom water, only Module3 was significant correlated with the pH and SAL (Figure 6C).

### 3.5 Observations on nitrogen function of bacterioplankton at various depths in water

**Figure 7:**
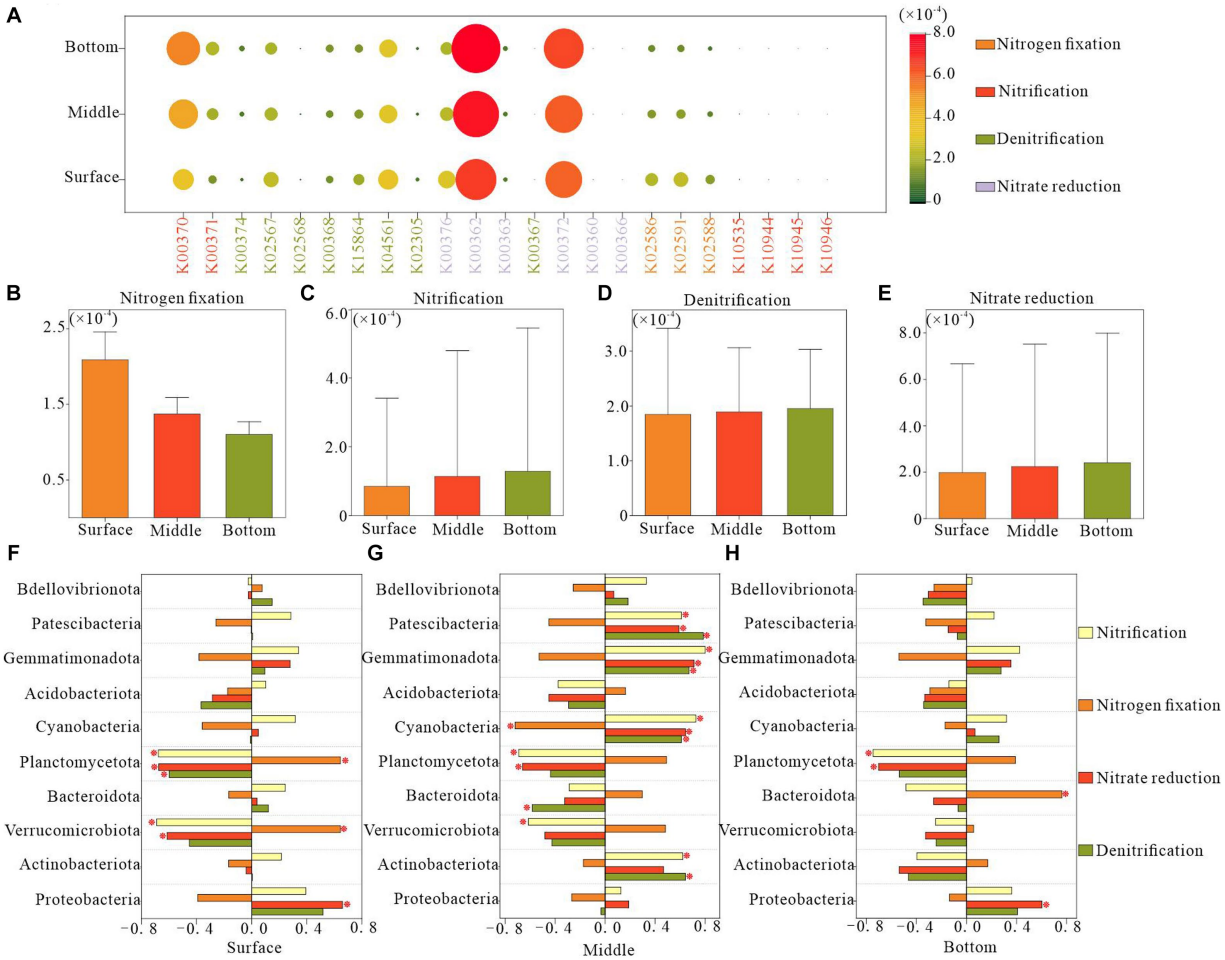
Functional genes of nitrogen metabolic pathways in bacterioplankton at different depths of the water column. **(A)** Relative abundance of various nitrogen functional genes, **(B–E)** relative abundance of different nitrogen metabolic process genes, and **(F–H)** correlation between the abundance of nitrogen functional genes and the dominant phyla in bacterioplankton.

Utilizing KEGG Ortholog groups (KOs) and Tax4Fun, we predicted potential nitrogen metabolism functional profiles for bacterial communities based on the 16S rRNA genes of the retrieved bacterial taxa. Our analysis focused on functional genes associated with nitrification, denitrification, nitrogen fixation, and nitrate reduction. We identified 23 potential nitrogen-related genes, with nitrate reduction genes, particularly K00362 and K00372, demonstrating the highest relative abundance across all functional pathways, followed by denitrification genes (Figure 7A). The surface layer exhibited a significantly higher relative abundance of potential genes for nitrogen fixation than did the bottom layer (Figure 7B). Conversely, potential genes for nitrification were less abundant in the surface layer than in the bottom layer (Figure 7C). The relative abundance of genes associated with denitrification and nitrate reduction remained relatively consistent across different water depths (Figures 7D,E).

In the surface water, the variation in the relative abundance of genes related to potential nitrogen metabolic functions in bacterioplankton was negatively correlated with the phyla Verrucomicrobiota and Planctomycetota and positively correlated with Proteobacteria. In the bottom layer, such correlations were primarily observed for Planctomycetota and Proteobacteria. The midwater bacterioplankton community exhibited the strongest association with nitrogen metabolic functions, specifically involving the phyla Planctomycetota, Cyanobacteria, Gemmatimonadota, and Patescibacteria. These findings indicated the pivotal role of these bacterioplankton taxa in the regulation of nitrogen metabolism (Figures 7F–H).

## 4 Discussion

Hulun Lake, a typical grassland inland lagoon, primarily receives recharge from atmospheric precipitation, groundwater, and rivers. During summer, increased rainfall contributes to more urban wastewater, animal manure, and hay entering the lake via rivers. In contrast, rising temperatures enhance the primary productivity of the lake, facilitating the release of bottom sediments. These seasonal variations have significantly altered the environmental conditions of the lake. In this study, we conducted a comprehensive analysis of the vertical variations in bacterioplankton community structure across different depths in Hulun Lake. Both exogenous inputs and endogenous releases affected the nutrient dynamics of the lake surface and bottom water environment, with the microbial community structure demonstrating variability at smaller taxonomic units, such as bacterial genera and OTUs. In addition, these alterations also affected the complexity of microbial network interactions. Our findings advanced the understanding of the ecological processes shaping this lake ecosystem.

### 4.1 Effects of exogenous inputs and endogenous releases on nutrient dynamics, microbial community composition, and function in the water

In this study, key environmental parameters such as pH, DO, COD, and various nitrogen forms exhibited significant depth-dependent variations. Surface water in direct contact with the atmosphere had higher DO levels, whereas exogenous inputs led to higher COD concentrations in surface water (Figure 2), which is consistent with our previous research (Wang, 2006; Zhao et al., 2007; Kumar et al., 2019; Nevers et al., 2020). The microbial community exhibited an evident depth-related succession pattern. Proteobacteria and Actinobacteriota emerged as the most dominant bacterial phyla in Hulun Lake, with the lowest relative abundances of 42.63 and 19.99% in surface water, and peak abundances of 45.17 and 26.25% in middle water, consistent with findings from other freshwater lakes (Christina et al., 2018) (Figure 4A; Supplementary Table S1). These shifts in bacterial community composition across water depths were attributed to inherent water characteristics.

Our study revealed that the bottom water possessed a higher diversity of bacterioplankton taxa (Figure 3B), which was likely influenced by two primary factors. First, the release of bottom sediments enriched the nutrient content of the bottom water, supporting bacterioplankton survival. Second, numerous studies have indicated that bottom sediments usually contain more abundant biological taxa than the water column, and frequent material-energy exchanges between these compartments further increase bacterioplankton diversity in bottom water (Perkins et al., 2014; Fang et al., 2015; Kumar et al., 2019; Sun et al., 2019; Khabouchi et al., 2020; Gao et al., 2022). Simultaneously, the spatial distribution of the sampling sites varied notably across different depths (Figure 3A). Surface water, influenced by factors such as atmospheric precipitation, wind speed, and river confluence, exhibits greater environmental instability and looser bacterioplankton distributions (Mesbah et al., 2007; Dong et al., 2019). In addition, the bacterioplankton community composition demonstrated significant variations in α-diversity across the surface, middle, and bottom layers (Figure 3D). We identified that physicochemical indicators, including pH and SAL, along with nutrient elements, such as TN, COD, and NH_4_^+^-N, were the key drivers of these microbial community changes (Supplementary Figure S1). However, TN and NH_4_^+^-N had stronger effects on the bacterioplankton community structure in the surface and benthic layers, respectively. pH was consistently correlated with bacterial communities at various depths, suggesting that both surface and bottom bacterioplankton communities were highly sensitive to nitrogen fluctuations, and slight variations in pH significantly affected the structure of the bacterial communities in Hulun Lake (Glassman et al., 2018; Wahdan et al., 2023).

The dominant bacterial taxa across different water depths remained consistent, predominantly Proteobacteria, Actinobacteriota, Verrucomicrobiota, and Bacteroidota at the phylum level. Additionally, *hgcI_clade*, *norank_f_Clade_III*, *CL500-29_marine_group*, *unclassified_f_Comamonadaceae*, and *norank_f_Pirellulaceae* were the most dominant at the genus level (Figures 4A,B), which is consistent with findings from other freshwater ecosystems (Newton et al., 2011; Shang et al., 2020). Nevertheless, notable variations in the relative abundances of these dominant genera were observed at different water depths, which are likely attributed to nutrient element fluctuations (Mesbah et al., 2007). A comparison of the NH_4_^+^-N content and the relative abundance of *norank_f_Pirellulaceae* revealed contrasting trends (Figure 2; Supplementary Table S2). RDA analysis confirmed a mutually inhibitory relationship between these variables across different depths (Supplementary Figure S1), suggesting that nutrient variations are key factors in bacterioplankton community structure (Feng et al., 2009; Fang et al., 2015; Qu et al., 2015). In the case of other dominant phyla such as *CL500-29_marine_group*, although their relative abundance changes paralleled those of NH_4_^+^-N, the relationship transitioned from mutual promotion to inhibition (Supplementary Figure S1). This indicated that the effects of nutrients on bacterial communities differ under different habitat conditions (Xiong et al., 2014; Liang et al., 2016; Huang et al., 2018).

Microbial community dynamics are crucial factors of community function and offer insights into ecophysiological responses to depth-dependent changes. Consistent with our expectations, strong correlations were observed between the nitrogen metabolic processes and microbial communities. Specifically, the relative abundance of genes related to nitrate reduction exhibited a positive correlation with Proteobacteria in both the surface and bottom layers but was negatively correlated with Planctomycetota. In contrast, Planctomycetota in the surface layer demonstrated a positive correlation with genes associated with nitrogen fixation and a negative correlation with nitrification-related genes in the middle and bottom layers (Figures 7F–H). This suggests that Proteobacteria promoted the depletion of NO_3_^−^-N in surface and bottom waters, as influenced by exogenous inputs and endogenous releases. Alternatively, sufficient contact between surface waters and the atmosphere facilitated an ample supply of N_2_, enabling microorganisms to promote nitrogen fixation, a process mainly dominated by Planctomycetota. In addition, we observed positive correlations between surface and bottom Proteobacteria and Planctomycetota with NH_4_^+^-N, whereas the middle-layer Planctomycetota was negatively correlated with NH_4_^+^-N (Supplementary Figure S1). These close associations between the dominant phyla and nitrogen suggest that the predominant phyla in the surface and bottom layers may promote nitrogen fixation and nitrate reduction. Conversely, the dominant phylum in the midwater may inhibit nitrification processes, thereby affecting NH_4_^+^-N levels at various depths in Hulun Lake (Figure 2). In a previous study, Fisher et al. (2000) reported that nutrient availability not only influenced microbial growth rates and productivity, but also significantly affected community composition and function, which is applicable to our study.

### 4.2 Influence of microbial interactions across depth-varying water masses and their environmental significance

Microorganisms exhibit complex ecological interactions that can be synergistic, reciprocal, or antagonistic (Shang et al., 2020). Recent studies have indicated a strong correlation between microbes in Hulun Lake and its rivers, driving distinct ecological functions (Shang et al., 2020). Our results suggest that increased lake depth corresponds to heightened complexity within the microbial network. Notably, the network structure of the mesocosm was more stable than those of the surface and bottom layers (Supplementary Table S4). This may be due to the frequent exchange of species energy between the surface and bottom bacterioplankton, which are more susceptible to environmental perturbations due to exogenous inputs and endogenous releases. In contrast, the midwater environment remains relatively stable (Jiao et al., 2016). Similar observations have been made in soil microbial communities, where improved microhabitat conditions influence the interactions between organisms (Li et al., 2013). Our results are supported by studies highlighting how environmental heterogeneity can engender variations in ecological network structure (Mitchell et al., 2010; Prober and Wiehl, 2011).

Proteobacteria, Bacteroidetes, and Actinobacteria were predominant in the microbial network structures across water samples at various depths (Supplementary Table S5). However, variations in the relative abundances of these dominant phyla across different samples and modules suggest diverse ecological roles they may play (Newman, 2006; Shang et al., 2020). Specifically, all modules in the surface water network were dominated by Proteobacteria, indicating their primary role in organic matter degradation (Chun et al., 2019; Shang et al., 2020; Zhang Y. H. et al., 2021). Moreover, our analysis revealed that environmental changes affected the bacterioplankton network structure not only at the phylum level but also significantly influenced key genera. Nanoarchaeota (*g_norank_f_GW2011_GWC1_47_15*), Verrucomicrobiota (*g_norank_f_cvE6*), and Bacteroidota (*g__unclassified_f_Balneolaceae*) emerged as the key bacterial taxa on the surface, mesopelagic, and bottom layers, respectively (Figures 5A–C). Despite their low abundance, these key taxa can substantially influence the biometabolic networks within the microbial community of the lake. Notably, the distribution of these keystone genera shifted from a single module in the surface waters to two modules in the bottom layer, suggesting potential fragmentation in their ecological interactions and functions (Figures 5D–F).

Significant differences were observed in the microbial communities across the surface, midwater, and bottom water. Notably, both the strength of module-environment variable correlations and modularity values were lower in the bottom water microbial communities than in the surface and mesocosm communities (Figures 6A–C; Supplementary Table S4). This suggests reduced connectivity between the bottom microbial communities. In the surface and midwater layers, the structures of the different ecological network modules, predominantly composed of Bacteroidota, Proteobacteria, and Actinobacteria, were mainly regulated by pH, DO, and TN (Figures 6A,B; Supplementary Table S5). These findings are consistent with existing literature, indicating that pH variations can reshape competitive relationships among microbial populations and induce changes in nutrient availability, phylum composition, and genus-level abundance (Fierer and Jackson, 2006; Lauber et al., 2009; Zhang H. G. et al., 2021). Furthermore, only module 3 in the bottom-water network exhibited a significant correlation with the environmental variables (Figure 6C). Collectively, our results suggest that microbial networks with higher connectivity and compactness have greater potential to be influenced by environmental factors.

## 5 Conclusion

Using 16S rRNA high-throughput sequencing technology, this study comprehensively assessed the bacterioplankton diversity in Hulun Lake. The findings demonstrated that both exogenous inputs and endogenous releases exerted a significant influence on bacterioplankton community structure. Specifically, the relative abundance of Proteobacteria and Actinobacteria increased from the surface to the bottom layers of the lake. Redundancy analysis further confirmed that habitat conditions significantly affected bacterioplankton composition. Among the assessed environmental factors, TN, pH, and COD were identified as the key variables influencing bacterioplankton communities in the surface, midwater, and bottom layers, respectively. Symbiotic network analysis suggested distinct relationships with water properties. However, because the mesocosms were less subject to exogenous inputs and endogenous releases, their microorganisms tended to survive independently, exhibiting fewer symbiotic relationships. Nevertheless, this structure might have more abundant ecological functions. Through the Tax4Fun analysis, 23 potential nitrogen-related functional genes were identified in the bacterioplankton community. The most significant functional roles were observed in nitrate reduction and denitrification processes. The flexibility of surface planktonic bacterial communities for nitrogen fixation was driven by exogenous input, whereas endogenous release led to similar flexibility in nitrification processes within the bottom planktonic communities. These results indicate the need for further metagenomic studies to validate the function of these genes. Overall, this study provides crucial insights into the complexity and functionality of bacterioplankton communities in Hulun Lake and emphasizes the importance of managing both surface- and bottom-water ecosystems through comprehensive control of external inputs and internal releases to improve lake ecology.

## Data availability statement

The original contributions presented in the study are included in the article/Supplementary material, further inquiries can be directed to the corresponding author.

## Author contributions

YS: Writing – original draft. WL: Writing – review & editing. XG: Writing – original draft.
