# Supplementary material for: Composition, interaction networks, and nitrogen metabolism patterns of bacterioplankton communities in a grassland type Lake: a case of Hulun Lake, China
Source: Front Microbiol. 2023 Nov 22;14:1305345. doi: 10.3389/fmicb.2023.1305345 (PMC10703169; doi:10.3389/fmicb.2023.1305345)
Supplement: Supplementary file 1 [file Data_Sheet_1.docx]

Supplementary Material

**Supplementary Table 1** Relative abundance of dominant phyla in water of different depths.

| phylum | surface water | | middle water | | bottom water | | p value |
| --- | --- | --- | --- | --- | --- | --- | --- |
|  | Mean(%) | Sd(%) | Mean(%) | Sd(%) | Mean(%) | Sd(%) |  |
| Proteobacteria | 42.630 | 14.790 | 43.710 | 13.480 | 45.170 | 13.890 | 0.8295 |
| Actinobacteriota | 19.990 | 8.067 | 24.490 | 8.733 | 26.250 | 7.189 | 0.2262 |
| Verrucomicrobiota | 9.770 | 7.679 | 8.047 | 6.943 | 5.281 | 3.588 | 0.5303 |
| Bacteroidota | 9.450 | 4.626 | 6.918 | 2.337 | 6.470 | 3.223 | 0.1064 |
| Planctomycetota | 8.310 | 7.568 | 7.671 | 6.073 | 4.798 | 3.148 | 0.5955 |
| Cyanobacteria | 4.809 | 2.916 | 3.175 | 1.641 | 3.782 | 3.713 | 0.2612 |
| Acidobacteriota | 1.778 | 1.278 | 2.237 | 1.845 | 1.844 | 1.357 | 0.8192 |
| Gemmatimonadota | 1.065 | 1.279 | 1.528 | 1.324 | 1.721 | 1.296 | 0.1771 |
| Patescibacteria | 0.440 | 0.479 | 0.5961 | 0.423 | 1.658 | 2.098 | 0.0145 |

**Supplementary Table 2** Relative abundance of dominant genus in water of different depths.

| genus | surface water | | middle water | | bottom water | | p value |
| --- | --- | --- | --- | --- | --- | --- | --- |
|  | Mean(%) | Sd(%) | Mean(%) | Sd(%) | Mean(%) | Sd(%) |  |
| *hgcI_clade* | 8.431 | 5.810 | 13.090 | 6.606 | 14.660 | 5.904 | 0.04366 |
| *norank_f__Clade_III* | 3.971 | 3.092 | 6.218 | 3.774 | 8.462 | 5.307 | 0.03415 |
| *CL500-29_marine_group* | 5.566 | 2.865 | 6.159 | 4.759 | 5.574 | 2.861 | 0.97140 |
| *unclassified_f__Comamonadaceae* | 5.057 | 3.274 | 5.805 | 3.279 | 4.373 | 2.531 | 0.53310 |
| *norank_f__Pirellulaceae* | 6.424 | 7.526 | 4.127 | 5.011 | 2.327 | 2.365 | 0.66510 |
| *Cyanobium_PCC-6307* | 4.165 | 2.875 | 2.791 | 1.654 | 2.815 | 2.148 | 0.35880 |
| *Rheinheimera* | 1.778 | 2.044 | 2.827 | 2.600 | 3.490 | 4.671 | 0.38270 |
| *LD29* | 4.119 | 4.131 | 2.731 | 3.415 | 1.117 | 0.836 | 0.21450 |
| *Flavobacterium* | 3.478 | 2.471 | 2.116 | 1.817 | 1.840 | 2.488 | 0.06106 |
| *norank_f__MWH-UniP1_aquatic_group* | 1.727 | 1.052 | 2.23 | 1.096 | 2.393 | 1.196 | 0.29960 |
| *Rhodobacter* | 4.297 | 6.843 | 1.219 | 1.287 | 0.635 | 0.859 | 0.00654 |
| *Pseudomonas* | 0.998 | 1.404 | 2.453 | 1.938 | 2.666 | 2.509 | 0.03098 |
| *Hydrogenophaga* | 1.624 | 1.002 | 2.183 | 2.106 | 2.046 | 1.718 | 0.99690 |
| *Vogesella* | 0.505 | 1.100 | 2.315 | 3.519 | 2.937 | 5.731 | 0.17880 |
| *Comamonas* | 3.060 | 8.457 | 1.483 | 3.406 | 0.470 | 0.696 | 0.98630 |
| *norank_f__67-14* | 1.631 | 1.583 | 1.165 | 0.985 | 1.389 | 1.262 | 0.70610 |
| *norank_f__norank_o__JG36-TzT-191* | 1.526 | 1.316 | 1.416 | 0.875 | 1.226 | 0.672 | 0.89210 |
| *Sphingorhabdus* | 1.917 | 2.001 | 1.067 | 1.365 | 0.826 | 1.303 | 0.08309 |
| *norank_f__norank_o__norank_c__OM190* | 0.764 | 0.890 | 1.873 | 1.747 | 1.164 | 1.294 | 0.05623 |
| *Terrimicrobium* | 1.396 | 1.703 | 1.573 | 2.512 | 0.789 | 1.215 | 0.45860 |
| *norank_f__Gemmatimonadaceae* | 0.912 | 1.198 | 1.292 | 1.200 | 1.540 | 1.229 | 0.14330 |
| *Acinetobacter* | 0.935 | 1.613 | 0.940 | 1.701 | 1.834 | 4.729 | 0.95470 |
| *Candidatus_Methylopumilus* | 0.985 | 0.801 | 1.127 | 0.444 | 1.333 | 0.545 | 0.28900 |
| *norank_f__Blastocatellaceae* | 1.151 | 1.142 | 1.481 | 1.760 | 0.661 | 0.601 | 0.35940 |
| *Novosphingobium* | 1.364 | 1.303 | 0.912 | 0.715 | 1.000 | 1.703 | 0.07046 |
| *unclassified_f__Rhodobacteraceae* | 1.202 | 0.740 | 1.010 | 0.678 | 0.800 | 0.772 | 0.21120 |
| *CL500-3* | 0.623 | 1.037 | 1.145 | 1.007 | 0.898 | 0.733 | 0.11440 |
| *unclassified_c__Actinobacteria* | 0.765 | 0.296 | 0.844 | 0.448 | 0.880 | 0.275 | 0.69520 |
| *Luteolibacter* | 1.613 | 2.158 | 0.624 | 1.155 | 0.195 | 0.350 | 0.01732 |
| *Candidatus_Planktophila* | 0.569 | 0.510 | 0.812 | 0.421 | 0.931 | 0.233 | 0.12920 |
| *unclassified_o__Burkholderiales* | 0.615 | 0.523 | 0.772 | 0.571 | 0.803 | 0.586 | 0.59780 |
| *Sediminibacterium* | 1.396 | 1.77 | 0.423 | 0.306 | 0.354 | 0.252 | 0.01199 |
| *norank_f__norank_o__norank* | 0.336 | 0.399 | 0.766 | 0.892 | 0.922 | 0.785 | 0.06149 |
| *Pseudohongiella* | 0.434 | 0.338 | 0.780 | 0.505 | 0.752 | 0.460 | 0.14060 |
| *Prosthecobacter* | 1.130 | 2.117 | 0.517 | 0.842 | 0.285 | 0.456 | 0.27630 |
| *norank_f__norank_o__Vicinamibacterales* | 0.476 | 0.472 | 0.574 | 0.609 | 0.695 | 0.624 | 0.59640 |
| *SH3-11* | 0.377 | 0.410 | 0.745 | 0.538 | 0.605 | 0.591 | 0.1120 |
| *Blastomonas* | 0.841 | 0.930 | 0.568 | 0.592 | 0.230 | 0.325 | 0.03851 |
| *Candidatus_Limnoluna* | 0.550 | 0.355 | 0.512 | 0.240 | 0.514 | 0.315 | 0.83760 |
| *Paracoccus* | 0.669 | 0.727 | 0.344 | 0.546 | 0.539 | 0.423 | 0.12770 |
| *Fluviicola* | 0.564 | 0.759 | 0.449 | 0.417 | 0.421 | 0.349 | 0.74870 |
| *Algoriphagus* | 0.442 | 0.456 | 0.546 | 0.821 | 0.439 | 0.357 | 0.87840 |
| *norank_f__norank_o__Kapabacteriales* | 0.493 | 0.615 | 0.420 | 0.349 | 0.507 | 0.325 | 0.47730 |
| *Polynucleobacter* | 0.328 | 0.269 | 0.418 | 0.332 | 0.501 | 0.450 | 0.68370 |
| *norank_f__NS11-12_marine_group* | 0.444 | 0.431 | 0.321 | 0.206 | 0.404 | 0.514 | 0.99720 |
| *Brevundimonas* | 0.414 | 0.236 | 0.236 | 0.222 | 0.513 | 1.228 | 0.08163 |
| *Dinghuibacter* | 0.372 | 0.303 | 0.472 | 0.327 | 0.301 | 0.189 | 0.44370 |
| *Arenimonas* | 0.294 | 0.311 | 0.490 | 0.518 | 0.336 | 0.419 | 0.56530 |
| *unclassified_f__Oxalobacteraceae* | 0.305 | 0.266 | 0.356 | 0.335 | 0.447 | 0.513 | 0.91010 |

**Supplementary Table 3** Relative abundance of dominant OTU in water of different depths.

| Species name | surface water | | middle water | | bottom water | | p value |
| --- | --- | --- | --- | --- | --- | --- | --- |
|  | Mean(%) | Sd(%) | Mean(%) | Sd(%) | Mean(%) | Sd(%) |  |
| OTU3163 | 3.971 | 3.092 | 6.218 | 3.774 | 8.462 | 5.307 | 0.03415 |
| OTU286 | 4.051 | 3.135 | 6.706 | 5.039 | 7.676 | 5.532 | 0.1771 |
| OTU996 | 2.391 | 2.208 | 3.861 | 2.248 | 4.17 | 1.944 | 0.04428 |
| OTU1425 | 3.034 | 2.295 | 4.061 | 3.002 | 2.641 | 1.844 | 0.4642 |
| OTU1039 | 2.818 | 2.143 | 3.641 | 3.837 | 2.92 | 1.971 | 0.9465 |
| OTU1070 | 3.583 | 6.549 | 0.6832 | 0.9285 | 0.3466 | 0.5862 | 0.0017 |
| OTU269 | 2.888 | 8.015 | 1.384 | 3.227 | 0.4366 | 0.653 | 0.9863 |
| OTU2905 | 3.01 | 3.552 | 1.935 | 2.663 | 0.9908 | 0.9883 | 0.6495 |
| OTU1034 | 2.763 | 2.695 | 1.986 | 2.342 | 0.8003 | 0.6277 | 0.2202 |
| OTU1487 | 0.4311 | 0.9265 | 2.051 | 3.158 | 2.551 | 4.909 | 0.1717 |
| OTU3166 | 0.768 | 1.033 | 2.014 | 1.662 | 1.895 | 1.995 | 0.05487 |
| OTU2710 | 1.47 | 1.048 | 2.091 | 2.137 | 1.934 | 1.715 | 0.9375 |
| OTU1603 | 1.361 | 0.7387 | 1.72 | 1.312 | 1.886 | 0.6272 | 0.1439 |
| OTU2686 | 1.241 | 1.264 | 1.699 | 1.509 | 1.919 | 2.03 | 0.6255 |
| OTU1661 | 0.7127 | 0.8492 | 1.772 | 1.679 | 1.085 | 1.232 | 0.05295 |
| OTU1037 | 1.289 | 1.615 | 1.405 | 2.423 | 0.6542 | 1.135 | 0.6287 |
| OTU3160 | 1.145 | 1.138 | 1.474 | 1.754 | 0.6446 | 0.5933 | 0.3313 |
| OTU2730 | 1.05 | 0.6877 | 1.406 | 0.7248 | 1.489 | 0.7255 | 0.2527 |
| OTU1475 | 1.173 | 1.73 | 1.161 | 1.771 | 1.348 | 1.398 | 0.8134 |
| OTU124 | 0.9748 | 0.7939 | 1.117 | 0.4371 | 1.323 | 0.5433 | 0.277 |

**Supplementary Table 4** Topological characteristics of the ecological network structure of bacterioplankton communities.

|  | surface water | middle water | bottom water |
| --- | --- | --- | --- |
| Average degree | 48.866 | 40.151 | 92.004 |
| Network diameter | 4.000 | 4.000 | 4.000 |
| Graph density | 0.098 | 0.080 | 0.184 |
| Modularity | 0.300 | 0.323 | 0.209 |
| Average clustering coefficient | 0.396 | 0.362 | 0.532 |
| Average path length | 2.205 | 2.183 | 1.938 |
| Edges | 12241 | 10078 | 23093 |

**Supplementary Table 5** Percentage of dominant phyla in the ecological network structure.

| Phylum | surface water (%) | middle water (%) | bottom water (%) |
| --- | --- | --- | --- |
| Proteobacteria | 0.370 | 0.386 | 0.346 |
| Bacteroidota | 0.136 | 0.116 | 0.108 |
| Actinobacteriota | 0.092 | 0.092 | 0.086 |
| Verrucomicrobiota | 0.072 | 0.070 | 0.072 |
| Patescibacteria | 0.058 | 0.072 | 0.062 |
| Firmicutes | 0.054 | 0.038 | 0.044 |
| Acidobacteriota | 0.030 | 0.028 | 0.032 |
| Bdellovibrionota | 0.026 | 0.028 | 0.028 |
| Cyanobacteria | 0.026 | 0.026 | 0.022 |
| Planctomycetota | 0.026 | 0.030 | 0.030 |
| Chloroflexi | 0.018 | 0.016 | 0.028 |
| Myxococcota | 0.016 | 0.018 | 0.016 |
| Dependentiae | 0.010 | 0.010 | 0.010 |
| Desulfobacterota | 0.010 | 0.008 | 0.006 |

**Supplementary Table 6** Percentage of module in the ecological network structure.

| Module | surface water (%) | middle water (%) | bottom water (%) |
| --- | --- | --- | --- |
| Module1 | 24.95 | 22.71 | 39.84 |
| Module2 | 18.96 | 16.33 | 31.67 |
| Module3 | 16.77 | 13.75 | 25.10 |
| Module4 | 14.77 | 12.95 | 2.58 |
| Module5 | 12.77 | 12.75 | 2.58 |
| Module6 | 11.58 | 12.35 | 2.58 |
| Others | 0.20 | 9.16 | 4.35 |


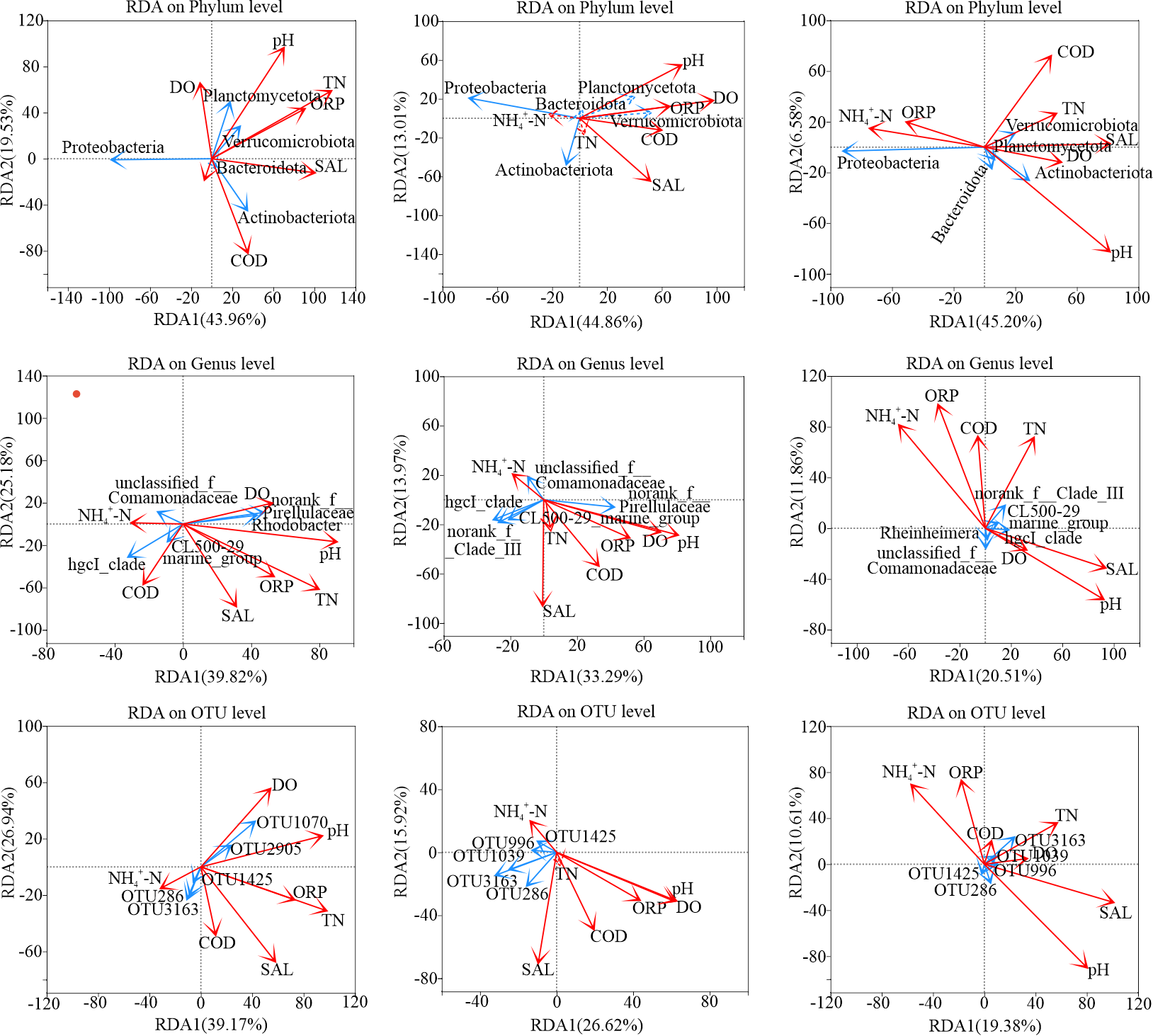


**Supplementary Figure 1** Redundancy analysis of bacterial phyla, genera, and OTUs in water of different depths with environmental variables.
